# Epidemiology of Extended-Spectrum β-Lactamase-Producing *E. coli* and Vancomycin-Resistant Enterococci in the Northern Dutch–German Cross-Border Region

**DOI:** 10.3389/fmicb.2017.01914

**Published:** 2017-10-05

**Authors:** Xuewei Zhou, Silvia García-Cobos, Gijs J. H. M. Ruijs, Greetje A. Kampinga, Jan P. Arends, Dirk M. Borst, Lieke V. Möller, Nicole D. Holman, Theo A. Schuurs, Lesla E. Bruijnesteijn van Coppenraet, Jan F. Weel, Jan H. van Zeijl, Robin Köck, John W. A. Rossen, Alexander W. Friedrich

**Affiliations:** ^1^University of Groningen, University Medical Center Groningen, Department of Medical Microbiology, Groningen, Netherlands; ^2^Laboratory of Medical Microbiology and Infectious Diseases, Isala Clinics, Zwolle, Netherlands; ^3^Department of Microbiology, CERTE Medische Diagnostiek en Advies, Groningen, Netherlands; ^4^Department of Intensive Care Medicine, Martini Hospital, Groningen, Netherlands; ^5^Centre for Infectious Diseases Friesland, Izore, Leeuwarden, Netherlands; ^6^Institute of Medical Microbiology, University Hospital Münster, Münster, Germany; ^7^Institute of Hospital Hygiene, Klinikum Oldenburg, Oldenburg, Germany

**Keywords:** WGS, cgMLST, VRE, ESBL, hospital, community, prevalence, cross-border research

## Abstract

**Objectives:** To reveal the prevalence and epidemiology of extended-spectrum β-lactamase (ESBL)- and/or plasmid AmpC (pAmpC)- and carbapenemase (CP) producing *Enterobacteriaceae* and vancomycin-resistant enterococci (VRE) across the Northern Dutch–German border region.

**Methods:** A point-prevalence study on ESBL/pAmpC/CP producing *Enterobacteriaceae* and VRE was carried out in hospitalized patients in the Northern Netherlands (*n* = 445, 2012–2013) and Germany (*n* = 242, 2012). Healthy individuals from the Dutch community (*n* = 400, 2010–2012) were also screened. In addition, a genome-wide gene-by-gene approach was applied to study the epidemiology of ESBL-*Escherichia coli* and VRE.

**Results:** A total of 34 isolates from 27 patients (6.1%) admitted to Dutch hospitals were ESBL/pAmpC positive and 29 ESBL-*E. coli*, three pAmpC-*E. coli*, one ESBL-*Enterobacter cloacae*, and one pAmpC-*Proteus mirabilis* were found. In the German hospital, 18 isolates (16 *E. coli* and 2 *Klebsiella pneumoniae*) from 17 patients (7.7%) were ESBL positive. In isolates from the hospitalized patients CTX-M-15 was the most frequently detected ESBL-gene. In the Dutch community, 11 individuals (2.75%) were ESBL/pAmpC positive: 10 ESBL**-***E. coli* (CTX-M-1 being the most prevalent gene) and one pAmpC *E. coli*. Six Dutch (1.3%) and four German (3.9%) hospitalized patients were colonized with VRE. Genetic relatedness by core genome multi-locus sequence typing (cgMLST) was found between two ESBL-*E. coli* isolates from Dutch and German cross-border hospitals and between VRE isolates from different hospitals within the same region.

**Conclusion:** The prevalence of ESBL/pAmpC-*Enterobacteriaceae* was similar in hospitalized patients across the Dutch–German border region, whereas VRE prevalence was slightly higher on the German side. The overall prevalence of the studied pathogens was lower in the community than in hospitals in the Northern Netherlands. Cross-border transmission of ESBL-*E. coli* and VRE seems unlikely based on cgMLST analysis, however continuous monitoring is necessary to control their spread and stay informed about their epidemiology.

## Introduction

International travel and patient care are risk factors for dissemination of bacteria including multidrug-resistant microorganisms (MDRO), such as extended-spectrum β-lactamase (ESBL) and carbapenemase (CP)-producing *Enterobacteriaceae* (Woerther et al., 2013; Muller et al., 2015), and vancomycin-resistant enterococci (VRE). The prevalence of the latter has increased in the last years due to successful polyclonal subpopulations of hospital-associated (HA) *Enterococcus faecium* [previously designated clonal complex (CC) CC17] and which are also associated with amoxicillin resistance (ARE) (Arias and Murray, 2012). These populations are distinct from *E. faecium* isolates in the community and isolates from non-human sources (Galloway-Pena et al., 2012; Lebreton et al., 2013).

Netherlands and Germany as bordering countries with possible transfer of patients between them, created a cooperative network to prevent the spread of MDRO and to harmonize guidelines in healthcare settings^1^ (Muller et al., 2015). Surveillance studies to monitor the prevalence, resistance patterns, and molecular background of MDRO in hospitals and the community are essential to get insights into their epidemiology to implement infection prevention measures. Bacterial whole-genome sequencing (WGS) has been demonstrated to be very useful for epidemiological surveillance and detection of antimicrobial resistance (Kwong et al., 2015). The gene-by-gene approach uses a defined set of genes to extract an allele-based profile which makes it scalable and portable between laboratories (Maiden et al., 2013; Leopold et al., 2014). A core genome multi-locus sequence typing (cgMLST) scheme has been developed for *E. faecium* to distinguish between epidemiologically related and unrelated isolates (de Been et al., 2015). Although there is no cgMLST scheme nor threshold publicly approved yet for *Escherichia coli*, there are several tools available that allow to define an *ad hoc* cgMLST.

The aim of this study was to perform a point-prevalence study on ESBL/plasmid-mediated AmpC (pAmpC)/CP-producing *Enterobacteriaceae* and HA *E. faecium* (VRE and ARE) in hospitals in the Northern Dutch–German border region and to determine the predominant resistance genes. In addition, stool community samples from the Northern Netherlands were screened for the same resistant pathogens. A cgMLST was used to study hospital and cross-border dissemination of ESBL-*E. coli* and VRE.

## Materials and Methods

### Study Design

A prospective point prevalence study was conducted in four of the largest hospitals (in total 3550 beds) in the Northern Netherlands between November 2012 and February 2013, covering a total population of approximately 2.85 million people. The Hospital Ethical Committee of the University Medical Center Groningen (UMCG) was informed and patients were approached to voluntarily participate in the study. Patients included in this study provided their written informed consent and a questionnaire concerning epidemiological and clinical data. The following high-risk wards for antibiotic-resistant microorganisms were selected: intensive care units (ICU), vascular surgery, internal medicine hematology/oncology, and dialysis wards (both for in- and outpatients). Gynecology and neurology (low-risk wards) were also included for comparison. From the largest German university hospital in the same (border) region, patients from four ICUs, a surgical ward, and a hematology/oncology ward were screened during October and November 2012 and included in the study. After consent agreement, all admitted patients from the studied wards were screened until completing a minimum of 100 samples per hospital.

The study in healthy people living in the Northern Netherlands was conducted retrospectively, using control patients included in a previous case–control study on microorganisms causing gastroenteritis. Control subjects were patients attending their general practitioner for a variety of medical questions, but no gastrointestinal problems, in the period between August 2010 and December 2012 (Bruijnesteijn van Coppenraet et al., 2015). No prevalence study was performed in the community in Germany.

### Sample Collection

A total of 445 rectal swabs (Copan ESwab^TM^) were taken from hospitalized patients (median age = 66 years, range 18–99 years) in the Northern Netherlands, 51.7% (*n* = 230) from men and 48.3% (*n* = 215) from women. A total of 328 (73.7%) patients were screened at high-risk wards and 117 (26.3%) patients were screened at low-risk wards (**Table 1**). In the German university hospital, 242 patients (median age = 64 years, range 0–94 years) were included, 64.5% (*n* = 156) men and 35.5% (*n* = 86) women. Of these 242 patients, 140 were screened only for ESBL, 22 only for VRE, and 80 for both. From the Dutch community study, 400 frozen faeces samples were included; 41% (*n* = 164) from men and 59% (*n* = 236) from women, 12% of the samples were from children. The median age of the healthy individuals was 47.5 years (range 0–84 years).

**Table 1:** Distribution of ESBL/pAmpC producing *Enterobacteriaceae*, and amoxicillin- and vancomycin-resistant *E. faecium* among the different wards in Dutch hospitals.

Ward	ESBL/pAmpC producing *Enterobacteriaceae*	Amoxicillin-resistant *E. faecium*	Vancomycin-resistant *E. faecium*
High-risk (*n* = 328)	19 (5.8%)	99 (30.2%)	6 (1.8%)
- Intensive care unit (*n* = 102)	6 (5.9%)	31 (30.4%)	1 (1%)
- Vascular surgery (*n* = 54)	6 (11.1%)	15 (27.8%)	1 (1%)
- Internal medicine hematology/oncology (*n* = 81)	1 (1.2%)	36 (44.4%)	2 (2.5%)
- Dialysis (*n* = 91)	6 (6.6%)	17 (18.7%)	2 (2.2%)
Low-risk (*n* = 117)	8 (6.8%)	6 (5.1%)	0 (0%)
- Gynecology (*n* = 55)	3 (5.5%)	1 (1.8%)	0 (0%)
- Neurology (*n* = 62)	5 (8.1%)	5 (8.1%)	0 (0%)
Total (*n* = 445)	27 (6.1%)	105 (23.6%)	6 (1.3%)

### Microbiological Detection, Identification, and Susceptibility Testing

#### Dutch Hospitals and Retrospective Dutch Community Study

Rectal swabs (Dutch hospitalized patients) and approximately 50 μg of feces per sample (Dutch community patients) were enriched in selective broths: VRA broth containing BHI (brain heart infusion) with 20 mg/L amphoterin-B, 20 mg/L aztreonam, 20 mg/L colistin, and 16 mg/L amoxicillin and TSB-VC broth containing tryptic soy broth with 8 mg/L vancomycin and 0.25 mg/L cefotaxime. Both broths were incubated for 24 h at 35 ± 1°C. Subsequently, 10 μL of VRA broth was subcultured on VRE Brilliance agar (Oxoid^®^) and BMEG-2 agar (blood agar containing 64 mg/L meropenem, 2 mg/L gentamicin, 10 mg/L oxacillin, and 20 mg/L amphotericin-B) for identification of VRE and all ARE, respectively. Ten microliters of TSB-VC broth was subcultured onto ME/CF/CX comparted plates, containing iso-sensitest agar with 1 mg/L meropenem, 1 mg/L ceftazidime, or 1 mg/L cefotaxime, respectively, plus 20 mg/L vancomycin and 20 mg/L amphotericin-B (Mediaproducts, Groningen), for selection of ESBL/pAmpC/CP-producing bacteria. Plates were incubated for 24 h at 35 ± 1°C, except for VRE Brilliance agar plates that were incubated for 48 h.

Suspected colonies on VRE Brilliance, BMEG-2 and ME/CF/CX agar plates were streaked on blood agar (one isolate per morphotype). Species identification was done by matrix-assisted laser desorption/ionization-time of flight mass spectrometry (MALDI-TOF MS) (Bruker Daltonik GmbH, Bremen). Confirmed *Enterococcus* spp. and *Enterobacteriaceae* spp., were tested for antibiotic susceptibility using VITEK^®^2 (bioMérieux) automatic system and EUCAST clinical breakpoints.

#### German Hospital

Rectal swabs were directly plated on chromID^®^ ESBL agar (bioMérieux) for ESBL screening and enriched Enterococcosel^TM^ Broth (Bile Esculin Azide Broth) (BD; Becton, Dickinson and Company) was used for VRE screening and subsequently cultured on chromID^®^ VRE agar (bioMérieux).

Species identification and antibiotic susceptibility testing was done by MALDI-TOF MS (Bruker Daltonik GmbH, Bremen) and VITEK^®^2 (bioMérieux), respectively, following EUCAST criteria. Confirmation of ESBL was performed using disk diffusion (cefotaxime 30 μg, cefotaxime 30 μg plus clavulanic acid 10 μg, ceftazidime 30 μg, ceftazidime plus clavulanic acid 10 μg, cefepime 30 μg, cefepime 30 μg plus clavulanic acid 10 μg, and cefoxitin 30 μg) (Mast Diagnostics, Bootle, UK).

### PCRs and Microarray

Enterococci isolates from Netherlands were screened by in-house PCR for *IS16* (a marker for specific hospital-associated strains), *vanA* and *vanB* genes as described previously (Clark et al., 1993; Werner et al., 2011). The GenoType Enterococcus (Hain Lifescience GmbH) was used in enterococci isolates from Germany, which detects species and genotypes *vanA*, *vanB*, *vanC1*, and *vanC2*. ESBL and VRE positive isolates were sent to our hospital for further characterization.

*Enterobacteriaceae* isolates resistant to third generation cephalosporins and natural chromosomal AmpC producers intermediate or resistant to cefepime were selected for DNA extraction using the UltraClean Microbial DNA Isolation Kit (Mo Bio Laboratories, Inc.) and further characterized for the presence of ESBL/AmpC genes using a DNA-array (Check-MDR CT103, Check-points, Wageningen, Netherlands) (Garcia-Cobos et al., 2015).

### Whole-Genome Sequencing of VRE and ESBL-*E. coli*

WGS was performed for all ESBL-*E. coli* and VRE isolates. For each isolate, several colonies (about 5 μL) of the culture were suspended in 300 μL microbead solution, which was subjected to DNA extraction with the Ultraclean Microbial DNA isolation kit (Mo Bio Laboratories, Carlsbad, CA, United States). The DNA concentration and purity were measured using a NanoDrop 2000c spectrophotometer (Thermo Fisher Scientific, Waltham, MA, United States) and the Qubit double-stranded DNA (dsDNA) HS and BR assay kits (Life Technologies, Carlsbad, CA, United States). One nanogram of bacterial DNA was used for library preparation. The DNA library was prepared using the Nextera XT library preparation kit with the Nextera XT v2 index kit (Illumina, San Diego, CA, United States). The library fragment length was aimed at fragments with a median size of 575 bases and was assessed with the Genomic DNA ScreenTape assay with the 2200 TapeStation system (Agilent Technologies, Waldbronn, Germany). Subsequently, the library was sequenced on a MiSeq sequencer, using the MiSeq reagent kit v2 generating 250-bp paired-end reads. Sequencing was aimed at a coverage of at least 60-fold. MiSeq data were processed with MiSeq control software v2.4.0.4 and MiSeq Reporter v2.4 (Illumina, San Diego, CA, United States). Reads were quality-trimmed using the CLC Genomics Workbench software version 9.0.1 (CLC bio, Aarhus, Denmark) using default settings except for the following modifications: “trim using quality scores was set to 0.02” and “discard reads below length was set to 15.” Subsequently, trimmed-reads were *de novo* assembled with an optimal word size of 29 and a minimum contig length of 500. Metrics on raw read and assembly level are provided in **Supplementary Table S1**.

### cgMLST of VRE and ESBL-*E. coli*

A genome-wide gene-by-gene comparison approach was used to determine the genetic relatedness using SeqSphere^+^ version 3.4.0 (Ridom GmbH, Münster, Germany) (Leopold et al., 2014).

Genome assemblies from the VRE isolates were analyzed using the *E. faecium* cgMLST scheme previously published, considering a cluster alert distance of 20 different alleles (de Been et al., 2015).

An *ad hoc* cgMLST and whole-genome MLST (wgMLST) scheme was determined for *E. coli* isolates using the MLST^+^ target definer function with default parameters^2^ and *E. coli* K-12 as a reference (GenBank accession no. NC_010473.1). The filters applied to reference genome were: “minimum length filter” that discards genes shorter than 50 bases; “start codon filter” that discards all genes that contain no start codon at the beginning of the gene: “stop codon filter” that discards all genes that contain no stop codon, more than one stop codon or if the stop codon is not at the end of the gene: “homologous gene filter” that discards all genes that have fragments that occur in multiple copies in a genome (with identity ≥90% and more than 100 bases overlap); “gene overlap filter” that discards the shorter of two overlapping flanking genes if these genes overlap >4 bp. The remaining genes were then used in a pairwise comparison using BLAST (Leopold et al., 2014) with 45 query genomes (**Supplementary Table S2a**). All genes of the reference genome that were common in all query genomes with a sequence identity of ≥90 and 100 overlap, and with the default parameter stop codon percentage filter turned on, formed the final cgMLST scheme; this discards all genes that have internal stop codons in >20% of the query genomes. Additionally, 26 plasmid sequences (**Supplementary Table S2b**) were added to exclude such genes are part of the cgMLST typing scheme. The final cgMLST scheme consisted of 1.771 targets/genes, and 2329 accessory genes were additionally included for the wgMLST scheme (**Supplementary Tables S3**, **S4**). The minimum coverage of the genome assemblies was 20 times (**Supplementary Table S1**) and the percentage of good targets/genes included in the cgMLST were 97.6% for *E. coli* and 98.6% for *E. faecium* (**Supplementary Tables S5**, **S6**).

Furthermore, to determine the genetic relatedness, the genetic distance for the *E. coli* isolates was calculated as the proportion of allele differences: dividing the number of allele differences between two genomes by the total number of genes commonly shared by those two genomes (Kluytmans-van den Bergh et al., 2016). In this study, thresholds for genetic distance were described to discriminate between epidemiologically related and unrelated *E. coli* isolates as 0.0095 when using wgMLST and 0.0105 for cgMLST.

*Escherichia coli* STs were determined uploading genome assemblies to SeqSphere+ software following the scheme of Wirth et al. (2006). Sequence genomes with no conclusive results for the 7-gene MLST were uploaded to the Enterobase database^3^. Additionally, *E. coli* major phylogenetic groups (A, B1, B2, and D) were analyzed *in silico* by using MLST^+^ Target Definer function of SeqSphere^+^, including the *chuA*, *yjaA*, and TSPE4.C2 loci (Clermont et al., 2000).

Genome assemblies were also uploaded to the Center for Genomic Epidemiology to extract information on resistance genes (ResFinder) and virulence factors (VirulenceFinder), and species confirmation for VRE and ESBL-*E. coli* (KmerFinder), and serotype (SerotypeFinder) and plasmid replicons (PlasmidFinder) for ESBL-*E. coli* (Zankari et al., 2012; Carattoli et al., 2014; Hasman et al., 2014; Joensen et al., 2014, 2015; Larsen et al., 2014).

### Statistical Analysis

In the Dutch hospital prevalence study, associations between ESBL and ARE carriage and the following variables were analyzed: length of hospital stay, antibiotic use, and (low- or high-risk) ward. Information was gathered by the questionnaires. Statistical analyses were performed using SPSS for Windows, v. 20.0. Univariate analyses were performed using the Fisher’s exact or Chi-square methods for categorical variables. The Mann–Whitney *U*-test was used as a non-parametric tests in variables with no normal distribution. Results with a *p*-value of ≤0.05 were considered to be statistically significant. All *p*-values are two-tailed.

## Results

### ESBL/pAmpC-Producing *Enterobacteriaceae*

Thirty-four isolates from 27 of the 445 included patients admitted to hospitals in the Northern Netherlands (6.1%) were confirmed ESBL and/or pAmpC positive. A total of 85.2% (23/27), 14.8% (4/27), and 3.7% (1/27) of these patients were positive for ESBL, pAmpC, and both, respectively. Among the 34 isolates, 32 were *E. coli*, of which 29 were ESBL positive and three were pAmpC producers. Resistance genes detected in the *E. coli* isolates are shown in **Table 2**. CTX-M-15 (*n* = 8) and CTX-M-14 (*n* = 8) were the most prevalent ones. The other two isolates were an *Enterobacter cloacae*, containing a CTX-M-1-like gene and a pAmpC CMY-II producing *Proteus mirabilis*. At high-risk wards, 19 patients (5.8%) were found with ESBL/pAmpC isolates compared to eight patients (6.8%) at low-risk wards (*p* = 0.68; NS). No association was found between ESBL/pAmpC carriage and antibiotic use, length of hospital stay or ward (**Table 1**).

**Table 2:** Molecular characterization of the *E. coli* isolates from the community and hospital patients in the Netherlands and Germany.

Sample^1^	Hospital/ward	β-Lactamase genes	Phylogroup	ST	CC
**Community**					
1_Esco_CA-NL		blaCTX-M-1, blaTEM-1B	B2	131	ST131
2_Esco_CA-NL		blaSHV-12	B2	117	None
3_Esco_CA-NL		blaCMY-2	D	2309	None
4_Esco_CA-NL		blaCTX-M-1	D	57	ST350
5_Esco_CA-NL		blaCTX-M-1, blaTEM-1B	A	10	ST10
6_Esco_CA-NL		blaCTX-M-1, blaTEM-1B	B1	1079	None
7_Esco_CA-NL		blaCTX-M-1, blaTEM-1B	A	10	ST10
8_Esco_CA-NL		blaCTX-M-15	D	648	ST648
9_Esco_CA-NL		blaCTX-M-15	A	617	ST10
10_Esco_CA-NL		blaCTX-M-15	A	1312	None
11_Esco_CA-NL		blaCTX-M-14b, blaTEM-1B	D	38	ST38
**Hospital**					
12_Esco_HA-NL	A/Gynecology	blaCTX-M-15, blaTEM-1B	D	5463	None
12b_Esco_HA-NL	A/Gynecology	blaCTX-M-15, blaTEM-1B	D	5463	None
13_Esco_HA-NL	A/Neurology	blaCTX-M-27	B2	131	ST131
14_Esco_HA-NL	A/Dialysis outpatient	blaCTX-M-15, blaTEM-1B	A	93	ST168
15_Esco_HA-NL	A/ICU	blaCMY-2, blaTEM-1B	D	354	ST354
16_Esco_HA-NL	A/ICU	blaCTX-M-15, blaTEM-1B, blaOXA-1	B1	58	ST155
17_Esco_HA-NL	A/ICU	blaCTX-M-15, blaTEM-1B	B1	38	ST38
18_Esco_HA-NL	A/ICU	blaTEM-52C	B1	453	ST86
19_Esco_HA-NL	A/ICU	blaCTX-M-1	B1	641	ST86
20_Esco_HA-NL	A/ICU	blaSHV-12	A	5888	None
20b_Esco_HA-NL	A/ICU	blaCTX-M-1	B1	58	ST155
21_Esco_HA-NL	B/Gynecology	blaCTX-M-14	B1	101	ST101
22_Esco_HA-NL	B/Dialysis outpatient	blaCTX-M-14	B1	38	ST38
22c_Esco_HA-NL	B/Dialysis outpatient	blaCTX-M-14	D	38	ST38
23_Esco_HA-NL	B/Vascular surgery	blaCMY-2, blaTEM-1B	D	1508	None
24_Esco_HA-NL	B/Neurology	blaTEM-52C	D	2064	None
25_Esco_HA-NL	B/Neurology	blaCTX-M-3, blaTEM-1B	B2	95	ST95
25b_Esco_HA-NL	B/Neurology	blaCTX-M-3, blaTEM-1B	D	95	ST95
26_Esco_HA-NL	C/Gynecology	blaCTX-M-15, blaOXA-1	B2	131	ST131
27_Esco_HA-NL	C/Dialysis outpatient	blaCTX-M-1, blaTEM-33	A	3478	None
28_Esco_HA-NL	C/Dialysis outpatient	blaCTX-M-14	A	10	ST10
29_Esco_HA-NL	C/Neurology	blaCTX-M-1	B1	603	None
30_Esco_HA-NL	C/Vascular surgery	blaCTX-M-14	A	410	ST23
31_Esco_HA-NL	D/Vascular surgery	blaCTX-M-14, blaTEM-1B, blaOXA-1	B1	58	ST155
32_Esco_HA-NL	D/Vascular surgery	blaCTX-M-1	D	117	None
32b_Esco_HA-NL	D/Vascular surgery	blaDHA-1, blaTEM-1B	B2	131	ST131
33_Esco_HA-NL	D/Vascular surgery	blaCTX-M-14	D	69	ST69
33b_Esco_HA-NL	D/Vascular surgery	blaCTX-M-14	D	69	ST69
34_Esco_HA-NL	D/Internal medicine	blaCTX-M-55, blaOXA-1	B1	4385	None
35_Esco_HA-NL	D/Dialysis outpatient	blaCTX-M-15, blaTEM-1B, blaOXA-1	B2	131	ST131
35b_Esco_HA-NL	D/Dialysis outpatient	blaCTX-M-15, blaOXA-1	B2	131	ST13
36_Esco_HA-NL	D/Dialysis outpatient	blaCTX-M-1, blaTEM-1B	B1	58	ST155
37_Esco_HA-DE	ICU 1	blaCTX-M-15	D	38	ST38
38_Esco_HA-DE	ICU 6	blaCTX-M-14	D	38	ST38
39_Esco_HA-DE	ICU 2	blaCTX-M-14	A	10	ST10
40_Esco_HA-DE	ICU 6	blaCTX-M-15, blaTEM-1B, blaOXA-1	B1	448	ST448
41_Esco_HA-DE	Surgical ward	blaCTX-M-1, blaTEM-1B	A	10	ST10
42_Esco_HA-DE	Hemato-oncology ward	blaCTX-M-15, blaTEM-1B, blaOXA-1	A	90	ST23
43_Esco_HA-DE	ICU 4	blaCTX-M-15, blaOXA-1	A	34	ST10
44_Esco_HA-DE	ICU 3	blaTEM-187	A	10	ST10
45_Esco_HA-DE	ICU 3	blaCTX-M-15, blaOXA-1	D	38	ST38
46_Esco_HA-DE	ICU 3	blaCTX-M-1, blaTEM-1B	A	10	ST10
47_Esco_HA-DE	ICU 1	blaCTX-M-15	D	38	ST38
48_Esco_HA-DE	ICU 1	blaCTX-M-14, blaTEM-1B	D	1177	–

^1^*CA, community acquired; HA, hospital acquired; NL, Netherlands; DE, Germany; numbers refer to individual patients and a letter behind a number indicates that more than one isolate was obtained from the patient*.

In the German hospital, a total of 18 isolates from 17 patients (17/220; 7.7%) were ESBL positive. Sixteen isolates were *E. coli* and two were *Klebsiella pneumoniae*. Of these, 12 *E. coli* and one *K. pneumoniae* isolates were available for molecular testing. Six out of 12 (50%) *E. coli* isolates and the *K. pneumoniae* isolate had a CTX-M-15 gene (**Table 2**).

In the retrospective Dutch community study, 11 patients (11/400; 2.75%) were ESBL/pAmpC positive: 10 ESBL-*E. coli* (CTX-M-1 being the most prevalent gene) and one pAmpC *E. coli* (**Table 2**). Overall, no carbapenem resistance was observed neither in the community nor in the hospitals.

### *Escherichia coli* MLST and Phylogenetic Groups

Among ESBL/pAmpC-*E. coli* isolates from Dutch hospitals, the most prevalent ST was ST131 (CC, ST131; *n* = 5, 15.6%), all of them belonging to phylogroup B2 (**Table 2**). In the Dutch community isolates, 10 different STs were found, most of them belonging to CC ST10 (*n* = 3, 27.3%) and one isolate to ST131 (phylogroup B2). In the German hospital, the most prevalent STs were ST38 (33.3%) and ST10 (33.3%) (**Table 2**).

### Amoxicillin- and Vancomycin-Resistant *E. faecium* (ARE and VRE)

In the Dutch hospitals, 105 patients (105/445; 23.6%) were colonized with ARE, including six patients (6/445; 1.3%) with VRE. All ARE were positive for *IS16* and all VRE were *vanB* positive. Colonization of ARE (and VRE) was associated with high-risk wards (*p* < 0.001), prolonged hospitalization (*p* < 0.001), and use of antibiotics (*p* = 0.05), especially penicillins and fluoroquinolones (*p* < 0.001) (**Table 3**).

**Table 3:** Variables associated with carriage of amoxicillin-resistant *E. faecium* (ARE) and extended-spectrum β-lactamase (ESBL)- and/or plasmid AmpC (pAmpC)- producing *Enterobacteriaecae*.

Variables	ARE (*n* = 105)	No ARE (*n* = 340)	*p*-Value^∗^	ESBL/pAmpC (*n* = 27)	No ESBL/pAmpC (*n* = 418)	*p*-Value^∗^
Hospitalization days median (range)	12 (1-127)	3 (1-107)	*p* < 0.001	4 (1-127)	4 (1-36)	*p* = 0.886
Ward			*p* < 0.001			*p* = 0.657
High-risk (*n* = 328)	99 (94.3%)	229 (67.4%)		19 (70.4%)	309 (73.9%)	
Low-risk (*n* = 117)	6 (5.7%)	111 (32.6%)		8 (29.6%)	109 (26.1%)	
Antibiotic use (*n* = 145)	62 (59%)	83 (24.4%)	*p* < 0.001	7 (25.9%)	138 (33%)	*p* = 0.529
Penicillins^∗∗^	26 (24.8%)	29 (8.5%)	*p* < 0.001	3 (11.1%)	35 (8.4%)	*p* = 0.494
Fluoroquinolones	28 (26.7%)	15 (4.4%)	*p* < 0.001	1 (3.7%)	42 (10%)	*p* = 0.499
Third generation cephalosporins	11 (10.5%)	19 (5.6%)	*p* = 0.081	1 (3.7%)	29 (6.9%)	*p* = 1.00

^∗^*Results with a *p*-value of ≤0.05 were considered to be statistically significant. All *p*-values are two-tailed. ^∗∗^Used penicillins: benzylpenicillin, flucloxacillin, amoxicillin–clavulanic acid, and piperacillin–tazobactam*.

In the border German university hospital four (4/102; 3.9%) VRE isolates were isolated. Three of them were *vanA* positive and one was *vanB* positive.

In the retrospective Dutch community study, six ARE (6/400; 1.5%) were found, three of them were *IS16* positive. Only one *vanA*-VRE (1/400; 0.25%) was found, this strain was amoxicillin susceptible and *IS16* negative.

### cgMLST and wgMLST Comparison of ESBL-*E. coli* Isolates from the Community and Hospitals

**FIGURE 1:**
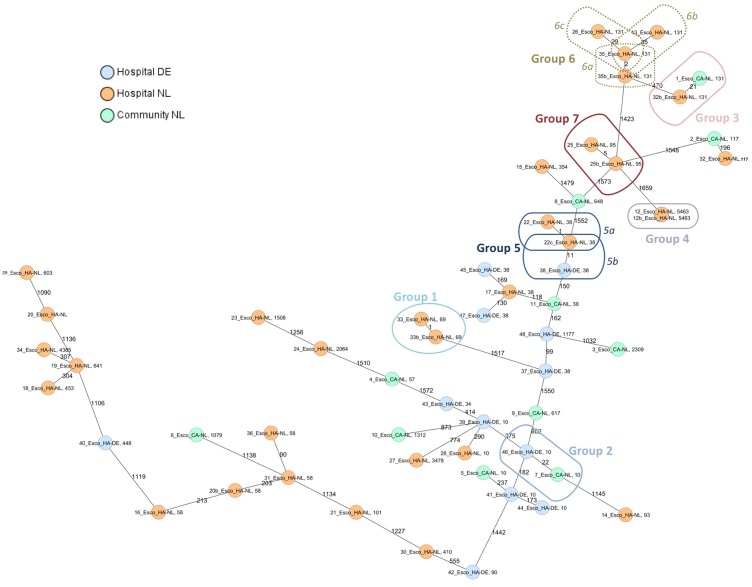
Minimum spanning tree of ESBL-*E. coli* isolates from hospitals and the community. Distance based on a cgMLST of 1771 genes using the parameters “pairwise ignoring missing values” during calculation. Each circle represents a genotype, colors indicate geographical origin and community or hospital. Orange: hospital—Netherlands; blue: hospital—Germany; green: community—Netherlands. Number of different alleles are indicated on the edges between connected isolates (nodes). The cut-off values for defining a group was 35 alleles. Isolates are presented by their ID and ST.

Genome assemblies of 55 ESBL-*E. coli* [Dutch community (*n* = 11), Dutch hospitals (*n* = 32), and German hospital (*n* = 12)] of this study were analyzed by a gene-by-gene approach and the allelic distance from the cgMLST and wgMLST were visualized in a minimum spanning tree (**Figure 1** and **Supplementary Figure S1**, respectively).

Six groups of isolates with a lower number of different alleles (≤35) by cgMLST were further analyzed. **Supplementary Table S7** summarizes the origin of the isolates in every group and the core and whole-genome genetic distance. Those groups formed by isolates with an epidemiological link (isolated from the same patient; group 1, 4, 5a, 6a, and 7), showed a core and whole-genome genetic distance lower than 0.0030 and 0.0046, respectively. In addition, isolates of group 5b, although with unknown epidemiological link, had a core genetic distance of 0.0063 and a whole-genome genetic distance of 0.0076. Both isolates were positive for CTX-M-14, however, no plasmid replicons were found in one of them (isolate 38_Esco_HA-DE) (**Supplementary Table S7**).

Among those groups including isolates with non (or unknown) epidemiological link, the core genome genetic distance was between 0.0122 and 0.0199 and the whole-genome genetic distance was between 0.0104 and 0.0208 (groups 2, 3, 6b, and 6c; **Figure 1**). Resistance and virulence profiles of the isolates are shown in **Supplementary Table S8**.

### cgMLST Comparison of VRE Isolates from the Community and Hospitals

**FIGURE 2:**
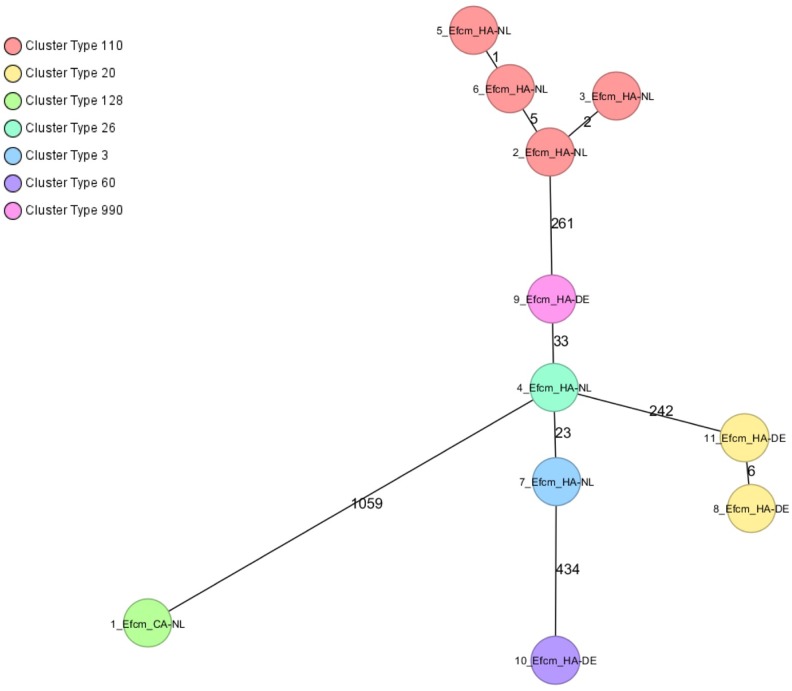
Minimum spanning tree of VREfcm, cgMLST based on 1423 genes using the parameters “pairwise ignoring missing values” during distance calculation. Each circle represents a genotype and colors indicate cluster types (CT). Number of different alleles are indicated on the edges between connected isolates (nodes). Isolates are presented by their ID, ST, and CT.

A minimum spanning tree was created for the 11 VRE isolates [Dutch community (*n* = 1), Dutch hospitals (*n* = 6), and German hospital (*n* = 4)]. Two clusters of isolates from different patients were observed (**Figure 2**). One cluster of four *vanB*-VRE isolates from the Dutch hospital belonged to cluster type (CT) 110 (ST17); two isolates were from the same ward in hospital A and the other two isolates were isolated from different wards in hospital B. The other cluster of two *vanA*-VRE isolates were isolated from different wards from the German hospital (CT 20, ST203). The resistance and virulence genotypes of VRE isolates are shown in **Supplementary Table S8**.

### Nucleotide Sequence Accession Number

Sequence data obtained in this study has been deposited at the National Center for Biotechnology Information under BioProject no. PRJNA352198.

## Discussion

This study shows the molecular epidemiology of ESBL/pAmpC-*E. coli* and HA *E. faecium* in hospitals in the Northern Dutch–German border region and the community in the Northern Netherlands. Dutch hospitals showed a prevalence for ESBL/pAmpC – *Enterobacteriaceae*, VRE, and ARE of 6.1, 1.3, and 23.6%, respectively, whereas the prevalence in the community was 2.75, 0.25, and 1.5%, respectively. The German hospital had an ESBL/pAmpC prevalence of 7.7 and 3.9% for VRE.

A previous study reported a prevalence of ESBL-producing bacteria of 4.9% in the Netherlands (Overdevest et al., 2014), comparable to the 6.1% prevalence observed in Dutch hospitals in this study. A prevalence of 5.6% ESBL-producing *E. coli* isolates in hospitalized and ambulatory patients in Germany has been reported recently (Pietsch et al., 2015), which is slightly lower than the 7.7% observed in the present study.

Furthermore, we observed an ESBL-*E. coli* prevalence of 2.5% in the Northern Netherlands community, which is low compared to previous studies in other regions, in which the prevalence in the community ranged from 4.7% (2009) to 10.1% (2011) (Reuland et al., 2013; van Hoek et al., 2015). This difference may have several reasons. First, ESBL prevalence may vary between regions and over time, and natural eradication of resistant *Enterobacteriaceae* might occur over time in the community (Bar-Yoseph et al., 2016). Additionally, samples included in this study were only chosen from patients without any gastrointestinal complaints, a factor which otherwise has been described to be associated with high ESBL prevalence (Reuland et al., 2013).

The majority of the resistance genes found in our community isolates were CTX-M-1 which is broadly disseminated among animals in Europe, especially in cattle and pigs, followed by the CTX-M-15 gene, commonly associated with human origin (Reuland et al., 2013; Pietsch et al., 2015). The latter was the most frequent gene among the Dutch and German hospital isolates, in concordance with previous studies (Ewers et al., 2012; Reuland et al., 2013; Pietsch et al., 2015).

The pAmpC prevalence in *E. coli* in our study was 0.3%, comparable to the prevalence of 0.6% what was reported in the study of van Hoek et al. (2015) (0.6% pAmpC *Enterobacteriaceae*) and somewhat lower to findings of Reuland et al. (2015) (1.3% pAmpC-*E. coli*). The most common pAmpC gene found in hospital and community isolates were CMY-II, which is together with DHA frequently found in human isolates (Reuland et al., 2015).

ESBL-producing *E. coli* belonging to CC ST131-phylogroup B2 are usually associated with more virulent strains (Overdevest et al., 2015). These were frequently found in the Dutch hospitals included in the present study but only sporadically in the community samples. This CC ST131-phylogroup B2 was also prevalent in a study carried out in hospitals in the Rotterdam region (van der Bij et al., 2011). CC ST10 was predominant among the ESBL-producing *E. coli* in the community, the same CC was also described to be prevalent in another Dutch study in community patients (Reuland et al., 2013).

We observed an overall ARE and VRE prevalence in hospitalized patients of 23.6 and 1.3%, respectively. Similar observations were made in a study performed in Dutch hospitals in 2008 reporting ARE carriage rates of 10–16% upon admission and 15–39% on acquisition in hematology and gastroenterology/nephrology wards (de Regt et al., 2008). The clinical significance of enterococcal infections and active VRE screening has been a matter of discussion. However, in immunocompromised patients, high morbidity and mortality rates have been reported in infections caused by enterococci (Zhou et al., 2013). In this study, ARE/VRE carriage was associated with prolonged hospitalization and antibiotic use, which is in line with previous literature (Torell et al., 1999). We found a high carriage rate of ARE in high-risk wards (30.2%). Notably, these patients may be at risk for a subsequent infection. Since 2011, VRE started to become a problem in multiple hospitals in the Netherlands: a total of 14 hospitals were affected with outbreaks of VRE in October 2012 (van den Brink, 2013). However, in this study a prevalence of VRE (*vanB*) carriage of only 1.3% was found. This is probably due to extensive infection prevention measures and successful outbreak management control. The prevalence of 1.3% is similar to what has been previously published in the Netherlands, with prevalence rates ranging from 1.4 to 2% in the 1990s (Endtz et al., 1997; van den Braak et al., 2000). The VRE prevalence in the German hospital was slightly higher (3.9%), though it is known that Germany has a higher VRE prevalence compared to the Netherlands^4^.

In our Dutch community one *vanA*-VRE was found, that was amoxicillin susceptible and *IS16* negative, indicative for a non-hospital origin (Galloway-Pena et al., 2012; Lebreton et al., 2013). Endtz et al. (1997) reported a higher number of VRE in the community (2%), however, this study did not include information about amoxicillin resistance nor *IS16* which makes it difficult to determine if they had a hospital or non-hospital origin (Galloway-Pena et al., 2012; Lebreton et al., 2013).

The cgMLST analysis in our study showed heterogeneity among *E. coli* species, and isolates were genetically distributed independently of their origin. The hospital and community ESBL-*E. coli* isolates included in this study did not show any genetic relatedness except for the ones isolated from the same patient and for two isolates (group 5b) from patients in different hospitals across the Dutch–German border, in a distance of approximately 200 km and with no known epidemiological link. The patient from the Dutch hospital was a dialyses outpatient (isolation date December 2012) whereas the patient from the German hospital was admitted to ICU (isolation date November 2012). Interestingly, both isolates harbored the same ESBL gene and virulence factors.

Genetic relatedness was found between four VRE isolates (CT110) from patients from two different Dutch hospitals (**Figure 2**), which indicates transmission between wards, but also between hospitals in a close geographical region similar to findings of a previous population-based study of VRE using WGS that also showed intra- and inter-regional spread of closely related VRE isolates (Pinholt et al., 2015). Although no genetic relatedness was found between VRE isolates of Dutch and German hospitals, the numbers of VRE isolates were too low to draw definite conclusions. It is known that several VRE CT co-circulate in Germany and the Netherlands (data not shown). However, only some laboratories have implemented the use of cgMLST in their routine to analyze VRE outbreaks and more epidemiological studies are needed to investigate cross-border transmission of VRE.

To our knowledge, there are no similar studies that compare and investigate the molecular epidemiology of ESBL-*E. coli* and VRE in hospitals and the community by WGS. Recently, the same approach has been used to study the clonality of ESBL-producing *Enterobacteriaceae* from environmental and stool samples from farmers suggesting possible cross-transmission between the farmers and the environment. This was only based on number of allele differences (Fischer et al., 2016) which makes it difficult to interpret results without considering the total number of genes included in the cgMLST scheme. In our study, we determined the genetic relatedness between ESBL-*E. coli* using cgMLST or wgMLST comparison and genetic distance calculation. These results were in concordance with the genetic distance thresholds of 0.0095 (wgMLST) and 0.0105 (cgMLST) previously established for *E. coli* based on known existing epidemiological links by analyzing more than 2000 ESBL-*Enterobacteriaceae* isolates from Dutch hospitals (Kluytmans-van den Bergh et al., 2016).

In another study, a cgMLST approach for several MDR bacteria was prospectively used for taking relevant infection control decisions in a hospital setting (Mellmann et al., 2016). A threshold of >10 differing alleles was defined to exclude nosocomial transmission of MDR *E. coli* (Mellmann et al., 2016). If we would have applied this threshold we would have missed the genetic relatedness between isolates belonging to group 5b, presenting 11 different alleles (**Figure 1** and **Supplementary Table S7**). This highlights that thresholds based on number of allele differences are only applicable to specific collections within a study, whereas the genetic distance calculation seems to give a more objective result, independently of the analyzed population.

We acknowledge this study has some limitations. No community study in the German cross-border region, neither ARE monitoring in the German hospital were performed. Laboratory methods for isolation of ESBL *Enterobacteriaceae* and VRE differed between Dutch and German hospitals since no enrichment broth was used in Germany, however, selective media agar was used in both regions. Since this study was anonymous, some epidemiological data were not available which makes it more difficult to draw conclusions regarding genetic relatedness among isolates between patients.

## Conclusion

In conclusion, the results of this study suggest that ESBL/pAmpC-*E. coli* circulate in the hospital and the community, although a higher prevalence of ESBL/pAmpC-*E. coli* was observed in hospitals compared to the community in the Northern Netherlands. Hospitals in the Northern Dutch–German region showed a similar prevalence of ESBL/pAmpC-*Enterobacteriaceae*. VRE prevalence was still low in the hospital as well as in the community in the Northern Netherlands. The German hospital showed a slightly higher VRE prevalence compared to hospitals in the Northern Netherlands. Nosocomial but no cross-border transmission of VRE was observed in this study. Epidemiologically related ESBL-*E. coli* and VRE were uncommon across the Northern Dutch–German border in the studied population, as only two ESBL-*E. coli* isolates from a Dutch and a German hospital were genetically similar. Cooperation between bordering countries and continuous monitoring using high discriminatory typing methods are still necessary to keep the epidemiology of resistant pathogens updated thereby helping to control their spread.

These results were partially presented at the ECCMID conference 2016, Amsterdam.

## Ethics Statement

The “METc (medical ethical committee) UMCG” believes that this research is not a research involving humans as is ment in the Law on Medical Scientific Research involving Human Beings (WMO). Therefore, the METc UMCG has decided that no WMO approval is needed.

## Author Contributions

GR, DB, LM, NH, TS, LB, JW, JvZ, and RK have all contributed to the collection of all samples, they have all reviewed the article carefully. XZ, SG-C, GK, JA, DB, JR, and AF have contributed to the study design and reviewed the article. XZ and SG-C wrote the manuscript together and analyzed the data. JR and AF reviewed the manuscript several times, before sending to the other authors for review.

## Conflict of Interest Statement

The authors declare that the research was conducted in the absence of any commercial or financial relationships that could be construed as a potential conflict of interest.

## Footnotes

1 https://www.Deutschland-Nederland.Eu

2 http://www.ridom.de/seqsphere/ug/v20/Tutorial_for_MLST+_Target_Definer.html

3 https://enterobase.Warwick.Ac.Uk

4 Antimicrobial Resistance Interactive Database (EARS-Net) (http://ecdc.Europa.eu/en/)

## Supplementary Material

FIGURE S1 Minimum spanning tree of ESBL-*E. coli* isolates from hospitals and the community. Distance based on a wgMLST of 4100 genes (cgMLST of 1771 genes and 2329 accessory genes) using the parameters “pairwise ignoring missing values” during calculation. Each circle represents a genotype, colors indicate geographical origin and community or hospital. Orange: hospital— Netherlands; blue: hospital—Germany; green: community—Netherlands. Number of different alleles are indicated on the edges between connected isolates (nodes). The same groups considered by cgMLST analysis are highlighted. Isolates are presented by their ID and ST.

TABLE S1 Metrics on raw read data and assemblies.

TABLE S2a Finished *E. coli* query genomes used in this study to develop and *ad hoc* cgMLST scheme (*n* = 45). One representative isolate of every ST from every collection community NL (*n* = 10), Dutch hospitals (*n* = 20) and German hospital (*n* = 6) of the present study and 9 *E. coli* genomes from Dutch patients and farmers previously published (de Been et al., 2014).

TABLE S2b Finished *plasmid* genomes for exclusion of genes with BLAST matches >90% and >100 bp length found within the query sequences used in this study to develop a cgMLST scheme.

TABLE S3 Genes included in the cgMLST scheme of *E. coli*.

TABLE S4 Accessory genes included in the wgMLST scheme of *E. coli*.

TABLE S5 *E. coli* cgMLST allele types for distance calculation and percentage of good targets/genes.

TABLE S6 *E. faecium* cgMLST allele types for distance calculation and percentage of good targets/genes.

TABLE S7 Genetic distance for pairwise comparisons of grouped ESBL- *E. coli* isolates.

TABLE S8 Results of ResFinder, VirulenceFinder, PlasmidFinder, and SerotypeFinder for *E. coli* and *E. faecium* isolates.
