# Supplementary figures and images for: Epidemiology of Extended-Spectrum β-Lactamase-Producing E. coli and Vancomycin-Resistant Enterococci in the Northern Dutch–German Cross-Border Region
Source: Front Microbiol. 2017 Oct 5;8:1914. doi: 10.3389/fmicb.2017.01914 (PMC5633748; doi:10.3389/fmicb.2017.01914)

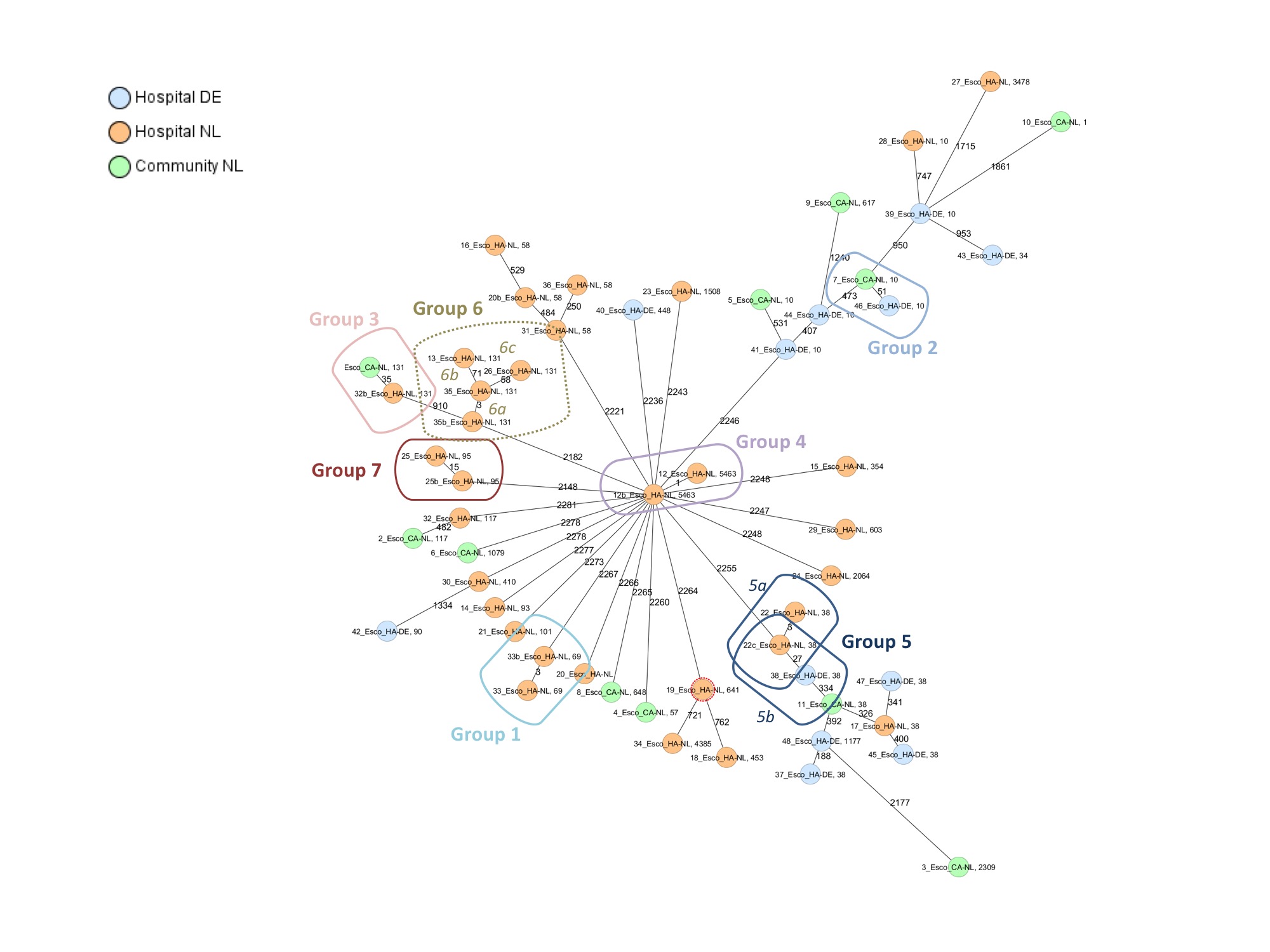

Supplement: FIGURE S1 — Minimum spanning tree of ESBL-E. coli isolates from hospitals and the community. Distance based on a wgMLST of 4100 genes (cgMLST of 1771 genes and 2329 accessory genes) using the parameters “pairwise ignoring missing values” during calculation. Each circle represents a genotype, colors indicate geographical origin and community or hospital. Orange: hospital— Netherlands; blue: hospital—Germany; green: community—Netherlands. Number of different alleles are indicated on the edges between connected isolates (nodes). The same groups considered by cgMLST analysis are highlighted. Isolates are presented by their ID and ST. [file Image_1.jpg]
