# Supplementary material for: Epidemiology of Extended-Spectrum β-Lactamase-Producing E. coli and Vancomycin-Resistant Enterococci in the Northern Dutch–German Cross-Border Region
Source: Front Microbiol. 2017 Oct 5;8:1914. doi: 10.3389/fmicb.2017.01914 (PMC5633748; doi:10.3389/fmicb.2017.01914)
Supplement: TABLE S2a — Finished E. coli query genomes used in this study to develop and ad hoc cgMLST scheme (n = 45). One representative isolate of every ST from every collection community NL (n = 10), Dutch hospitals (n = 20) and German hospital (n = 6) of the present study and 9 E. coli genomes from Dutch patients and farmers previously published (de Been et al., 2014). [file Table_2.DOCX]

Table S2.a. Finished *E.coli* query genomes used in this study to develop and *ad hoc* cgMLST scheme (n=45). One representative isolate of every ST from every collection (community NL (n=10), Dutch hospitals (n=20) and German hospital (n=6) of the present study and 9 *E. coli* genomes from Dutch patients and farmers previously published (de Been et al. 2014)

| **Strain** | **Source** | **Place of isolation** | **BioSample. No.** | **Ref.** |
| --- | --- | --- | --- | --- |
| 148 | Human (blood) | Utrecht | SAMN02471499 | De Been *et al* |
| 320 | Human (urine) | Utrecht | SAMN02471480 | De Been *et al* |
| 1350 | Human (urine) | Leeuwarden | SAMN02471497 | De Been *et al* |
| 1365 | Human (urine) | Leeuwarden | SAMN02471498 | De Been *et al* |
| 597 | Human (urine) | Groningen | SAMN02471510 | De Been *et al* |
| 606 | Human (pulmonary) | Groningen | SAMN02471485 | De Been *et al* |
| FAH1 | Human (faeces) | farm A | SAMN02471475 | De Been *et al* |
| FBH1 | Human (faeces) | farm B | SAMN02471517 | De Been *et al* |
| FCH1 | Human (faeces) | farm | SAMN02471511 | De Been *et al* |
| 1_Esco_CA-NL | Human | Community - NL | SAMN05967539 | This study |
| 2_Esco_CA-NL | Human | Community - NL | SAMN05977321 | This study |
| 3_Esco_CA-NL | Human | Community - NL | SAMN05977322 | This study |
| 4_Esco_CA-NL | Human | Community - NL | SAMN05977323 | This study |
| 5_Esco_CA-NL | Human | Community - NL | SAMN05977324 | This study |
| 6_Esco_CA-NL | Human | Community - NL | SAMN05977325 | This study |
| 8_Esco_CA-NL | Human | Community - NL | SAMN05977327 | This study |
| 9_Esco_CA-NL | Human | Community - NL | SAMN05977328 | This study |
| 10_Esco_CA-NL | Human | Community - NL | SAMN05977329 | This study |
| 11_Esco_CA-NL | Human | Community - NL | SAMN05977330 | This study |
| 12_Esco_HA-NL | Human | Hospital - NL | SAMN05977331 | This study |
| 13_Esco_HA-NL | Human | Hospital - NL | SAMN05977333 | This study |
| 14_Esco_HA-NL | Human | Hospital - NL | SAMN05977334 | This study |
| 15_Esco_HA-NL | Human | Hospital - NL | SAMN05977335 | This study |
| 16_Esco_HA-NL | Human | Hospital - NL | SAMN05977336 | This study |
| 17_Esco_HA-NL | Human | Hospital - NL | SAMN05977337 | This study |
| 18_Esco_HA-NL | Human | Hospital - NL | SAMN05977338 | This study |
| 19_Esco_HA-NL | Human | Hospital - NL | SAMN05977339 | This study |
| 20_Esco_HA-NL | Human | Hospital - NL | SAMN05977340 | This study |
| 21_Esco_HA-NL | Human | Hospital - NL | SAMN05977342 | This study |
| 23_Esco_HA-NL | Human | Hospital - NL | SAMN05977345 | This study |
| 24_Esco_HA-NL | Human | Hospital - NL | SAMN05977346 | This study |
| 25_Esco_HA-NL | Human | Hospital - NL | SAMN05977347 | This study |
| 27_Esco_HA-NL | Human | Hospital - NL | SAMN05977350 | This study |
| 28_Esco_HA-NL | Human | Hospital - NL | SAMN05977351 | This study |
| 29_Esco_HA-NL | Human | Hospital - NL | SAMN05977352 | This study |
| 30_Esco_HA-NL | Human | Hospital - NL | SAMN05977353 | This study |
| 32_Esco_HA-NL | Human | Hospital - NL | SAMN05977355 | This study |
| 33_Esco_HA-NL | Human | Hospital - NL | SAMN05977357 | This study |
| 34_Esco_HA-NL | Human | Hospital - NL | SAMN05977359 | This study |
| 37_Esco_HA-DE | Human | Hospital - DE | SAMN05977363 | This study |
| 39_Esco_HA-DE | Human | Hospital - DE | SAMN05977365 | This study |
| 40_Esco_HA-DE | Human | Hospital - DE | SAMN05977366 | This study |
| 41_Esco_HA-DE | Human | Hospital - DE | SAMN05977367 | This study |
| 42_Esco_HA-DE | Human | Hospital - DE | SAMN05977368 | This study |
| 43_Esco_HA-DE | Human | Hospital - DE | SAMN05977369 | This study |

*de Been, M., V. F. Lanza, M. de Toro, J. Scharringa, W. Dohmen, Y. Du, J. Hu, et al. 2014. Dissemination of cephalosporin resistance genes between escherichia coli strains from farm animals and humans by specific plasmid lineages. PLoS Genetics 10 (12) (Dec 18): e1004776.*

Table S2.b. Finished *plasmid* genomes for exclusion of genes with BLAST matches >90% and >100bp length found within the query sequences used in this study to develop a cgMLST scheme.

| **Strain** | **Plasmid** | **GenBank Acc. No.** | **Ref.** |
| --- | --- | --- | --- |
| *Escherichia coli* 042 | pAA | NC_017627.1 |  |
| *Escherichia coli* APEC O1 | pAPEC-O1-R | NC_009838.1 |  |
| *Escherichia coli* ETEC H10407 | p948 | NC_017724.1 |  |
| *Escherichia coli* JJ1886 | pJJ1886_5 | NC_022651.1 |  |
| *Escherichia coli* O104:H4 str. 2009EL-2050 | p09EL50 | NC_018651.1 |  |
| *Escherichia coli* O104:H4 str. 2011C-3493 | pESBL-EA11 | NC_018659.1 |  |
| *Escherichia coli* O111:H- str. 11128 | pO111_1 | NC_013365.1 |  |
| *Escherichia coli* O127:H6 str. E2348/69 | pE2348-2 | NC_011602.1 |  |
| *Escherichia coli* O157:H7 EDL933 | pO157 | NC_007414.1 |  |
| *Escherichia coli* O157:H7 str. TW14359 | pO157 | NC_013010.1 |  |
| *Escherichia coli* O157:H7 str. Sakai | pO157 | NC_002128.1 |  |
| *Escherichia coli* O26:H11 str. 11368 | pO26_1 | NC_013369.1 |  |
| *Escherichia coli* O55:H7 str. CB9615 | pO55 | NC_013942.1 |  |
| *Escherichia coli* O55:H7 str. RM12579 | p12579_1 | NC_017653.1 |  |
| *Escherichia coli* O7:K1 str. CE10 | pCE10A | NC_017647.1 |  |
| *Escherichia coli* O83:H1 str. NRG 857C | pO83_CORR | NC_017659.1 |  |
| *Escherichia coli* PMV-1 | pHUSEC411like | NC_022371.1 |  |
| *Escherichia coli* SE11 | pSE11-1 | NC_011419.1 |  |
| *Escherichia coli* SE15 | pECSF1 | NC_013655.1 |  |
| *Escherichia coli* UM146 | pUM146 | NC_017630.1 |  |
| *Escherichia coli* UMN026 | p1ESCUM | NC_011749.1 |  |
| *Escherichia coli* UMNK88 | pUMNK88 | NC_017645.1 |  |
| *Escherichia coli* UTI89 | pUTI89 | NC_007941.1 |  |
| *Escherichia coli* W | pRK1 | NC_017637.1 |  |
| *Escherichia coli* W | pRK1 | NC_017665.1 |  |
| *Escherichia coli* Xuzhou21 | pO157 | NC_017907.1 |  |
