# Supplementary material for: Epidemiology of Extended-Spectrum β-Lactamase-Producing E. coli and Vancomycin-Resistant Enterococci in the Northern Dutch–German Cross-Border Region
Source: Front Microbiol. 2017 Oct 5;8:1914. doi: 10.3389/fmicb.2017.01914 (PMC5633748; doi:10.3389/fmicb.2017.01914)
Supplement: TABLE S7 — Genetic distance for pairwise comparisons of grouped ESBL- E. coli isolates. [file Table_7.DOCX]

Table S7. Genetic distance for pairwise comparisons of grouped ESBL- *E. coli* isolates.

|  |  |  |  |  |  | **genetic distance** | |
| --- | --- | --- | --- | --- | --- | --- | --- |
| **Sample ID** | **ST** | **Phylogroup** | **Origin** | **Ward** | **Groups** | **cgMLST** | **wgMLST** |
| 33_Esco_HA-NL | 69 | D | HA-NL | Vascular surgery | group 1 | 0,0006 | 0,0008 |
| 33b_Esco_HA-NL | 69 | D | HA-NL | Vascular surgery |  |  |  |
| 7_Esco_CA-NL | 10 | A | CA-NL | **-** | group 2 | 0,0124 | 0,0135 |
| 46_Esco_HA-DE | 10 | A | HA-DE | ICU |  |  |  |
| 1_Esco_CA-NL | 131 | B2 | CA-NL | **-** | group 3 | 0,0122 | 0,0104 |
| 32b_Esco_HA-NL | 131 | B2 | HA-NL | Vascular surgery |  |  |  |
| 12_Esco_HA-NL | 5463 | D | HA-NL | Gynaecology | group 4 | 0 | 0,0004 |
| 12b_Esco_HA-NL | 5463 | D | HA-NL | Gynaecology |  |  |  |
| 22_Esco_HA-NL | 38 | B1 | HA-NL | Dialysis outpatient | group 5a | 0,0006 | 0,0008 |
| 22c_Esco_HA-NL | 38 | D | HA-NL | Dialysis outpatient | group 5a /5b |  |  |
| 38_Esco_HA-DE | 38 | D | HA-DE |  | group 5b | 0,0063 | 0,0076 |
| 35b_Esco_HA-NL | 131 | B2 | HA-NL | Dialysis outpatient | group 6a | 0,0012 | 0,0009 |
| 35_Esco_HA-NL | 131 | B2 | HA-NL | Dialysis outpatient | group 6a / 6b / 6c |  |  |
| 13_Esco_HA-NL | 131 | B2 | HA-NL | Neurology | group 6b | 0,0199 | 0,0208 |
| 26_Esco_HA-NL | 131 | B2 | HA-NL | Gynaecology | group 6c | 0,0165 | 0,0170 |
| 25_Esco_HA-NL | 95 | B2 | HA-NL | Neurology | group 7 | 0,0030 | 0,0046 |
| 25b_Esco_HA-NL | 95 | B2 | HA-NL | Neurology |  |  |  |

HA: hospital acquired; CA: community acquired; NL: The Netherlands; DE: Germany
